# Transfusion-dependent non-severe aplastic anemia: characteristics and outcomes in the clinic

**DOI:** 10.3389/fimmu.2023.1197982

**Published:** 2023-07-11

**Authors:** Yawen Zhang, Yao He, Suli Wang, Jingnan Sun, Jinsong Jia, Yuemin Gong, Guangsheng He, Jianyong Li

**Affiliations:** ^1^ Department of Hematology, The First Affiliated Hospital of Nanjing Medical University, Jiangsu Province Hospital, Collaborative Innovation Center for Cancer Personalized Medicine, Nanjing, China; ^2^ Department of Hematology, The Second People’s Hospital of Lianyungang, Lianyungang, China; ^3^ Department of Hematology, Shanghai Jiao Tong University Affiliated Sixth People’s Hospital, Shanghai, China; ^4^ Department of Hematology, The First Bethune Hospital of Jilin University, Changchun, China; ^5^ Department of Hematology, Peking University People’s Hospital, Peking University Institute of Hematology, Beijing, China

**Keywords:** transfusion-dependent, non-severe, aplastic anemia, antithymocyte globulin, cyclosporin A, immunosuppressive therapy

## Abstract

Transfusion-dependent non-severe aplastic anemia (TD-NSAA) is a rare condition of bone marrow failure that can persist for a long time or develop into severe aplastic anemia (SAA). Little is known about the clinical and laboratory characteristics, and disease prognosis and outcomes in TD-NSAA patients. The clinical and laboratory data of 124 consecutive TD-NSAA patients in the Chinese Eastern Collaboration Group of Anemia from December 2013 and January 2017 were analyzed retrospectively. In 124 TD-NSAA patients, the median age was 32 years (range: 3-80) and the median disease course was 38 months (range: 3-363). Common complications were iron overload (53/101, 52.5%), liver and kidney dysfunction (42/124, 33.9%), diabetes mellitus/impaired glucose tolerance (24/124, 19.4%), and severe infection (29 cases, 23.4%). 58% of patients (57/124) developed severe aplastic anemia with a median progression time of 24 months (range: 3-216). Patients with absolute neutrophil count (ANC) <0.5×10^9^/L, severe infection, or iron overload had a higher probability of progression to SAA (P=0.022, P=0.025, P=0.001). Patients receiving antithymocyte globulin (ATG) plus Cyclosporin A (CsA) had a higher overall response rate compared to those receiving CsA alone (56.7% vs 19.3%, P < 0.001). The addition of ATG was the favorable factor for efficacy (P=0.003). Fourteen patients developed secondary clonal hematologic disease: eleven patients with paroxysmal nocturnal hemoglobinuria, two patients with myelodysplastic syndromes, and one patient with acute myeloid leukemia, respectively. Ten patients (8.1%) died with a median follow-up of 12 months (range: 3- 36 months). Patients with TD-NSAA usually have a prolonged course of disease, and are prone to be complicated with important organ damage and disease progression to SAA. Intensive immunosuppressive therapy based on ATG might be an appropriate approach for TD-NSAA.

**Clinical trial registration:** http://www.chictr.org.cn/edit.aspx?pid=125480&htm=4, identifier ChiCTR2100045895.

## 1 Introduction

Aplastic anemia (AA) is a bone marrow failure disorder and presents as hypocellular bone marrow and peripheral blood pancytopenia (1). AA is caused by immune destruction mediated through T cells, resulting in severe deficiency of hematopoietic stem cells (2, 3). Severe AA (SAA) and non-severe AA (NSAA) were defined according to the severity of cytopenia (4), showing distinct outcomes in the disease prognosis, as well as the therapy choices. Based on blood transfusion requirements, NSAA can be further divided into two subtypes, transfusion-dependent NSAA (TD-NSAA) and transfusion-independent NSAA. Multiple studies have paid attention to the diagnosis and therapies of SAA (1, 5, 6), and immunosuppressive therapy has been widely used in SAA (7, 8). There were few large-scale clinical studies on TD-NSAA (9), and the natural course and appropriate therapy of TD-NSAA remain undetermined. In this study, we aim to describe the clinical characteristics and outcomes of TD-NSAA and explore effective treatments of TD-NSAA through retrospective analysis of patients registered in the China Eastern Anemia Cooperation Group (CECGA) (*ChiCTR2100045895*).

## 2 Materials and methods

### 2.1 Clinical data

The clinical and laboratory data of 124 patients with TD-NSAA in the CECGA: the First Affiliated Hospital of Nanjing Medical University/Jiangsu Province Hospital (Nanjing, China), the Second People’s Hospital of Lianyungang (Lianyungang, China), the South Campus of Shanghai Jiao Tong University Affiliated Sixth People’s Hospital, the First Hospital of Jilin University (Changchun, China), Peking University People’s Hospital (Beijing, China) from December 2013 to January 2017 were collected retrospectively. Demographic data, bone marrow aspirate smears and pathological reports, blood routine tests, biochemical tests, virological indicators, infection-related indicators, and treatment regimens were collected and analyzed. The eligible patients were followed until April 2017, and the median time to follow-up was 12 months (range 3-39). This study was approved by the participating hospitals’ ethical committee.

### 2.2 Inclusion criteria

TD-NSAA was defined as AA patients whose marrow cellularity < 25% (or 25–50% with <30% residual hematopoietic cells) and met at least one of the following criteria: absolute neutrophil count (ANC) < 0.5×10^9^/L; blood platelet count (BPC) < 20×10^9^/L; absolute reticulocyte count (ARC)< 20×10^9^/L, but not meet the criteria of severe aplastic anemia (10), with red blood cell transfusion ≥ 2U monthly or platelet transfusion to prevent bleeding.

Patients with congenital bone marrow failure (Fanconi anemia, Diamond Blackfan anemia, and dyskeratosis congenital), clinically classic paroxysmal nocturnal hemoglobinuria (PNH) (detected by FLAER method by flow), myeloproliferative neoplasms, myelodysplastic syndrome (MDS), and acute myeloid leukocyte (AML) were excluded. Two patients with classical PNH at baseline, one with Fanconi anemia and one with dyskeratosis congenita were excluded.

### 2.3 Treatment regimens and response evaluation criteria

CsA was administrated orally at an initial dose of 4-6 mg/kg daily and adjusted appropriately based on CsA concentrations or side effects, with concentrations between 150 and 200 μg/L. Rabbit anti-human thymocyte immunoglobulin (ATG) was administered intravenously for 5 consecutive days at a dose of 3.5 mg (/kg×d) in combination with CSA.

The responses were evaluated 6 months after CsA or ATG plus CsA treatments. Also, we evaluated the responses of all patients at the last follow-up time. Complete response (CR) is defined as an ANC > 1.0×10^9^/L, hemoglobin > 100 g/L, BPC >100×10^9^/L, and not requiring transfusion. Patients requiring transfusions after treatment are attributed to non-remission (NR). Partial response (PR) is a condition between CR and NR and does not meet the diagnostic criteria of AA.

Routine peripheral blood examinations including white blood cell count, ANC, absolute lymphocyte count (ALC), hemoglobin level, ARC and BPC, bone marrow aspirate and biopsy, chromosomes, immunophenotype, the function of vital organs and adverse events of TD-NSAA patients were analyzed retrospectively. Iron overload was defined as serum ferritin > 1000 μg/L, excluding elevated ferritin caused by active inflammation, liver disease, tumors, hemolysis, and alcoholism. Complications and adverse events were evaluated according to Common Terminology Criteria for Adverse Events version 4.0 (11).

### 2.4 Statistics

Summary statistics were used to describe patient characteristics, response, and laboratory parameters. Statistical Package for Social Science version 20.0 was used for statistical analyses. Median ± standard deviation was used for continuous variables and proportion for qualitative variables. Mann-Whitney U test was used to compare medians of two groups, while the Chi-square test and Fisher’s exact test were applied to qualitative data. The relevant risk factors were analyzed by binary logistic regression analysis. P-value < 0.05 was considered to be statistically significant.

## 3 Results

### 3.1 General information

From December 2013 to January 2017, 201 AA patients were enrolled in the CECGA. 124 (61.7%) TD-NSAA patients were involved in the final analysis, including 66 (53.2%) males and 58 (46.8%) females, with a median age of 32 years (range: 3–80) and a median disease course of 38 months (range: 3–363). There were 19 patients under the age of 18 years old, and 105 patients were over 18 years old (**Table 1**). Of all TD-NSAA patients, the progressive group and non-progressive group were classified according to whether the patients developed SAA. Patients who developed SAA showed lower BPC and ANC compared to those in the non-progressive group (P = 0.04, P = 0.06). Notably, a higher proportion of patients in the progressive group exhibited higher ALC, anemic heart disease, diabetes or impaired glucose tolerance, iron overload, and severe acute infection than patients who did not develop SAA (P=0.036, P=0.012, P=0.006, P=0.001, P<0.001) (**Table 1**).

**Table 1:** Baseline characteristics of TD-NSAA.

Projects	Progressive group (n=57)	Non-progressive group (n=67)	Statistic	P value
Age (Median)	32 (3~80)	32 (7~80)	t=0.190	0.346
Disease course (Median)	64 (8~362)	32 (3~363)	t=-1.970	0.1
Gender (Male/Female)	28/29	38/29	χ^2 ^= 0.713	0.398
ANC (×10^9^/L, median)	0.82 (0.05~7.85)	1.15 (0.24~3.26)	t=1.884	0.06
ALC (×10^9^/L, median)	1.11 (0.36-3.93)	1.51 (0.18~4.18)	t=0.998	0.036
ARC (×10^9^/L, median)	24.92 (3.81~134.8)	45.1 (3.7~379.2)	t=2.757	0.7
BPC (×10^9^/L, median)	13 (1~102)	17 (2~177)	t=1.764	0.04
Complications				
Liver and kidney dysfunction	20 (35.1%)	22 (32.8%)	χ^2 ^= 0.070	0.792
Gingival hyperplasia	0 (0%)	6 (8.9%)	χ^2 ^= 5.346	0.006
Anemic heart disease	9 (15.7%)	3 (4.5%)	χ^2 ^= 4.509	0.012
Hypertension	3 (5.2%)	4 (5.9%)	χ^2 ^= 0.029	0.865
Diabetes or impaired glucose. tolerance	17 (29.8%)	7 (10.4%)	χ^2 ^= 7.408	0.006
Iron overload	37 (64.9)	16 (23.9%)	χ^2 ^= 21.187	0.001
Severe infection	22 (38.6%)	7 (10.4%)	χ^2 ^= 13.620	0
Oral ulcer or herpes	3 (5.2%)	2 (3.0%)	χ^2 ^= 0.413	0.52
Viral hepatitis	17 (29.8%)	21 (31.3%)	χ^2 ^= 0.033	0.855
Death	7 (12.2%)	3 (4.5%)	χ^2 ^= 2.529	0.112
MDS/AML	1 (1.7%)	2 (3.0%)	χ^2 ^= 0.198	0.657
PNH	4 (7.0%)	7 (10.4%)	χ^2 ^= 0.488	0.503

ANC, absolute neutrophil count; ALC, absolute lymphocyte count; ARC, absolute reticulocyte count; BPC, blood platelet count; MDS, myelodysplastic syndrome; AML, acute myeloid leukocyte; PNH, paroxysmal nocturnal hemoglobinuria.

### 3.2 Complications

Liver and kidney dysfunction (42 cases, 33.9%), viral hepatitis (29 cases, 23.4%, hepatitis A in 1 case, and hepatitis B in 28 cases), severe infection (29 cases, 23.4%), diabetes or impaired glucose tolerance (24 cases, 19.4%), hypertension (7 cases, 5.6%), and oral ulcer or herpes (5 cases, 4.0%) were the common complications among all TD-NSAA patients. 53 patients (52.5%) exhibited iron overload in 101 patients assessed for iron load, and 12 patients (11.9%) were complicated with anemic heart disease, with a median developing time of 31 months (range: 8–314). Gingival hyperplasia occurred in 6 of 97 patients (6.2%) treated with CsA as a side effect.

### 3.3 Disease prognosis

During the follow-up, 57 patients (46.0%) developed SAA, with a median progression time of 24 (range: 3–216) months. The median time to progression from diagnosis was 24 months (range: 3-216) in 50 adults (47.6%), and 44 months (range: 6-70) in 7 children (36.8%). We then analyzed the risk factors associated with progression based on significant differences between progressive and non-progressive groups in **Table 1**, as well as risk factors that might be related to disease occurrence and development. Multivariate analysis revealed that low ANC (ANC < 0.5 × 10^9^/L, P=0.025), severe infection (P=0.022), and iron overload (P=0.001) were associated with the progression. Nevertheless, the course of disease (P= 0.365), BPC < 20 × 10^9^/L (P=0.300), ALC (P=0.407), ARC < 20×10^9^/L (P=0.284), diabetes or impaired glucose tolerance (P=0.608), and anemic heart disease (P=0.793) showed no significant effects on the disease progression (**Table 2**).

**Table 2:** Multivariate analysis of clinical factors related to the progression of SAA.

Factors	RR	95%CI	P value
Disease course	1.003	0.997~1.009	0.365
Iron overload	5.408	1.995~14.656	0.001
ANC<0.5×10^9^/L	4.758	1.247~18.160	0.022
ALC<1.0×10^9^/L	0.627	0.208~1.889	0.407
BPC<20×10^9^/L	1.744	0.610~4.985	0.300
ARC<20×10^9^/L	0.561	0.195~1.615	0.284
Severe infection	3.882	1.185~12.331	0.025
Diabetes or impaired glucose tolerance	0.725	0.213~2.471	0.608
Anemic heart disease	1.263	0.221~7.216	0.793

ANC, absolute neutrophil count; ALC, absolute lymphocyte count; ARC, absolute reticulocyte count; BPC, blood platelet count.

Clonal evolution was assessed in all patients. 11 patients (8.9%) developed PNH with a median time of 12.5 (range: 3–36) months, including 6 classic PNH subjects and 5 subjects with PNH with bone marrow disease - AA. Two patients converted to MDS - refractory cytopenia with multilineage dysplasia (RCMD) at 26 and 120 months, respectively, and one patient developed into AML at 22 months. A total of 14 subjects were transformed into clonal diseases and no significant clinical characteristics related to clonal disease conversion through univariate and multivariate analyses (**Table 3**).

**Table 3:** Multivariate analysis of clinical factors related to clonal progression.

Factors	RR	95%CI	P value
Disease course	1.000	0.989~1.011	0.994
Iron overload	0,966	0.924~1.010	0.132
Age	1.256	0.349~4.515	0.727
Gender (Male)	0.745	0.165~3.371	0.702
ANC<0.5×10^9^/L	0.352	0.040~3.104	0.347
ALC<1.0×10^9^/L	1.349	0.374~8.406	0.470
BPC<20×10^9^/L	2.117	0.396~11.329	0.381
ARC<20×10^9^/L	1.423	0.326~6.211	0.639

ANC, absolute neutrophil count; ALC, absolute lymphocyte count; BPC, blood platelet count; ARC, absolute reticulocyte count.

### 3.4 Efficacy

Among 119 patients (96.0%) who initially received CsA treatment, the overall response rate (ORR) was 19.3% (23 cases). And the ORR was 20% (3/15) in children and 19.2% (20/104) in adults. Of all 30 patients treated with a combination of CsA and ATG (5 subjects with TD-NSAA and 25 subjects with SAA in the progressive group), the ORR was 56.7% (17/30), which is much higher compared to that of CsA monotherapy (56.7% vs 19.3%; P < 0.001). There were no significant differences in the ORR between 5 TD-NSAA patients receiving ATG plus CSA treatments and patients receiving CsA alone (60% vs 19.3%; P=0.062). However, the ORR of 25 patients who progressed to SAA receiving ATG plus CsA treatment was 56%, which was markedly higher than patients treated with CsA alone (56% vs 28.1%; P < 0.001). Correspondingly, the ORR of ATG combined with CsA therapy in children and adults were 50% (4/8) and 59% (13/22), respectively, which were also higher than that of CsA monotherapy respectively (50% vs. 20%; P=0.182, 59% vs. 19.2%; P=0.0003). Four patients treated with a combination of CsA and ATG relapsed, and one patient relapsed after CsA monotherapy. Five patients received androgen monotherapy, and only one patient achieved PR. Two patients underwent hematopoietic cell transplant (HCT) during the follow-up and one obtained CR.

We then analyzed the relevant factors relating to efficacy. Multivariate analysis revealed that age (P=0.716), course of disease (P=0.504), iron overload (P=0.317), ANC < 0.5×10^9^/L (P=0.999), ALC (P=0.144), BPC < 20×10^9^/L (P=0.138) and ARC < 20×10^9^/L (P=0.684) showed no significant influence on the response. Importantly, the addition of ATG was a favorable factor for efficacy (P=0.003) (**Table 4**).

**Table 4:** Multivariate analysis of clinical factors related to efficacy.

Factors	RR	95%CI	P value
Age	1.019	0.992~1.046	0.716
Disease course	0.998	0.992~1.004	0.504
ANC<0.5×10^9^/L	1.000	0.340~2.948	0.999
ALC<1.0×10^9^/L	0.450	0.154~1.313	0.144
BPC<20×10^9^/L	0.492	0.193~1.256	0.138
ARC<20×10^9^/L	1.238	0.443~3.463	0.684
Iron overload	0.625	0.249~1.568	0.317
Treatments (ATG or CSA)	0.211	0.075~0.593	0.003

ANC, absolute neutrophil count; ALC, absolute lymphocyte count; ARC, absolute reticulocyte count; BPC, blood platelet count.

### 3.5 Survival

There were 10 deaths (8.1%) with a median follow-up of 12 months (range: 3- 36). Two patients died after ATG, one case died from pneumonia after undergoing transplantation and the other two cases died from intracranial hemorrhage. In addition, one patient died of advanced multiple organ failure due to pancreatic malignancy, one patient died of acute myocardial infarction, and one patient died of hemoptysis due to pneumonia. Two others died for unknown reasons.

Two deaths occurred in the patients treated with ATG in combination with CsA (6.67%), and the other 8 deaths were patients treated with CSA alone (8.99%). However, there were no significant differences in mortality between the above treatments. Gender, age, ANC < 0.5×10^9^/L, ALC < 1.0×10^9^/L, PLT < 20×10^9^/L, ALC < 20×10^9^/L, disease course, disease progression, iron overload, and treatments did not impact the all-cause death by univariate and multivariate analyses (**Table 5**).

**Table 5:** Multivariate analysis of clinical factors related to death.

Factors	RR	95%CI	P value
Gender (Male)	0.36	0.064-2.015	0.245
Age	1.048	0.999-1.100	0.053
BPC<20×109/L	1.938	0.305-12.321	0.483
ALC<1.0×10^9^/L	1.116	0.220-5.667	0.895
ANC<0.5×10^9^/L	1.519	0.273-8.465	0.633
ARC<20×109/L	2.023	0.402-10.183	0.393
Iron overload	1.673	0.321-8.718	0.541
Disease progression	1.171	0.197-6.945	0.862
Disease course	1.000	0.991-1.008	0.990
Treatments (ATG or CSA)	0.454	0.068-3.023	0.414

ANC, absolute neutrophil count; ALC, absolute lymphocyte count; BPC, blood platelet count; ARC, absolute reticulocyte count.

## 4 Discussion

At present, there were few systematic studies on TD-NSAA, and the clinical characteristics and risk factors associated with disease progression remain unclear. In our study, male and female showed a similar prevalence rate of TD-NSAA. Patients were generally young, but a median course of 38 months indicated patients with TD-NSAA usually have a prolonged course of the disease. Iron overload, liver and kidney dysfunction, diabetes or impaired glucose tolerance, and severe infection were common complications in TD-NSAA patients.

The Chinese guidelines for the treatment of TD-NSAA recommend CsA as front-line therapy, and the therapeutic schedule of SAA can be considered when the treatment fails at 6 months (12). Nishio et al. (13) reported the natural course of 22 children with transfusion-independent NSAA. Only 45% of children could maintain transfusion-independent with supportive treatment, whereas over half of children (55%) progressed into transfusion-dependent NSAA or SAA, with a five-year progression-free survival rate of 62 ± 12%. Kwon et al. (14) reported the natural course and prognosis of NSAA in 96 adults. Among them, 40 cases were asymptomatic at initial diagnosis, and 56 cases (58.3%) had typical symptoms. 34 patients did not receive any treatment, while the other 62 patients were treated with androgen, CsA, and ATG in combination with CsA, respectively. 18 cases (18.8%) progressed to SAA, with a median time of 18 months during the follow-up.

Although it showed no significant differences in ORR in TD-NSAA patients receiving ATG compared with those receiving CsA alone, due to the limited sample size, all patients treated with ATG including progressed to SAA were found to have a higher ORR. Multivariate analysis also revealed that ATG treatment was the only favorable factor associated with efficacy. It was suggested that early intervention with ATG plus CSA treatment might be better than with CSA monotherapy in TD-NSAA. The research of the European Cooperative Group for Bone Marrow Transplantation (EBMT) SAA working party also indicated that ATG combined with CsA was superior to CsA alone in ORR (74% vs. 46%) (9). Based on this, we speculated that the prolonged course of illness of TD-NSAA (median course of 38 months) may be due to a high proportion of patients using CsA treatment (96.0%), while the efficacy is poor. Moreover, 46.0% of TD-NSAA patients developed SAA with a median time of 24 months (range: 3–216 months) in this study. The research of EMBT has found that fewer patients in ATG combined with the CSA group progressed to SAA compared with those in the CsA group of NSAA patients (14.8% vs. 29.5%) (9). Therefore, it is necessary to recommend ATG combined with CsA as the front-line therapy for TD-NSAA. Moreover, Zhang et al. reported that the 5-year overall survival rate was 91.8% in NSAA pediatric patients after HCT, which suggested HCT could be a safe and effective treatment for TD-NSAA patients (15).

To explore risk factors associated with SAA progression, we performed multiple analyses in our cohort. ANC < 0.5×10^9^/L (P=0.025), severe infection (P=0.022), and iron overload (P=0.001) were associated with SAA progression. ANC < 0.5×10^9^/L was one of the diagnostic criteria of SAA, which indicates serious damage of the myeloid, resulting in an elevated risk of serious infection and the aggravation of bone marrow failure. Iron overload is caused by more iron absorption and less iron excretion after anemia, and excessive iron storage in the body after blood transfusion, owing to no physiological mechanism for excreting excess iron in human organism (16). Iron overload could reflect the degree of anemia, as well as ineffective erythropoiesis indirectly, and could cause various consequences such as heart failure and arrhythmias, liver fibrosis, and endocrine disorders (16, 17). Kwon et al. (14) also reported that the decreased count of white blood and ANC were risk factors for the progression of TD-NSAA. For TD-NSAA patients with ANC < 0.5×10^9^/L, severe infection, and iron overload, ATG might be chosen as the first-line treatment. Also, allogeneic hematopoietic stem cell transplantation could be considered a possible improvement in the prognosis.

In the previous study, Kwon et al. (14) found that 3 of 96 adult patients with NSAA developed secondary hematologic disease, one each for AML, MDS, and PNH. In our study, 11 patients (8.8%) developed PNH, which is higher than the incidence reported by Kwon et al. (14). The probability of a clone evolving into MDS (2 cases), and AML (1 case) is similar between our study and Kwon et al. (14). No pre-treatment clinical characteristics were found associated with clonal transformation.

However, the cohort size of the current study was small. In addition, we also need to extend follow-up, especially to study the risk factors related to the progression to SAA and the occurrence of clonal evolution.

As mentioned above, TD-NSAA is characterized by early disease onset and multiple complications with poor response to CsA alone. Meanwhile, patients with iron overload, lower ANC, and infection are prone to progressing to SAA. Early application of ATG combined with CsA may be an appropriate approach for TD-NSAA patients and remains worthy of exploration via further studies.

## Data availability statement

The raw data supporting the conclusions of this article will be made available by the authors, without undue reservation.

## Ethics statement

The studies involving human participants were reviewed and approved by The First Affiliated Hospital of Nanjing Medical University Ethic Committee. Written informed consent to participate in this study was provided by the participants’ legal guardian/next of kin.

## Author contributions

GH conceived the study. YH, SW, JS, and JJ collected the clinical and laboratory data. YZ, YG, GH and JL assisted with data interpretation. YZ, YH and GH wrote the manuscript with the help of all the authors. All authors contributed to the article and approved the submitted version.
